# A hypothermia mimetic molecule (zr17-2) reduces ganglion cell death and electroretinogram distortion in a rat model of intraorbital optic nerve crush (IONC)

**DOI:** 10.3389/fphar.2023.1112318

**Published:** 2023-01-23

**Authors:** Daniela S. Contartese, Manuel Rey-Funes, Rafael Peláez, Manuel Soliño, Juan C. Fernández, Ronan Nakamura, Nicolás S. Ciranna, Aníbal Sarotto, Verónica B. Dorfman, Juan J. López-Costa, José M. Zapico, Ana Ramos, Beatriz de Pascual-Teresa, Ignacio M. Larrayoz, César F. Loidl, Alfredo Martínez

**Affiliations:** ^1^ Departamento de Biología Celular, Histología, Embriología y Genética, Instituto de Biología Celular y Neurociencia “Prof. E. De Robertis” (IBCN), UBA-CONICET, Facultad de Medicina, Universidad de Buenos Aires, Buenos Aires, Argentina; ^2^ Biomarkers and Molecular Signaling, Neurodegenerative Diseases Area, Center for Biomedical Research of La Rioja (CIBIR), Logroño, Spain; ^3^ Centro de Estudios Biomédicos Básicos, Aplicados y Desarrollo (CEBBAD), Universidad Maimónides, Buenos Aires, Argentina; ^4^ Department of Chemistry and Biochemistry, Facultad de Farmacia, Universidad San Pablo-CEU, CEU Universities, Madrid, Spain; ^5^ Angiogenesis Group, Center for Biomedical Research of La Rioja, Logroño, Spain

**Keywords:** traumatic optic neuropathy, therapeutic hypothermia, hypothermia mimetics, intravitreal injection, electroretinogram, apoptosis

## Abstract

**Introduction:** Ocular and periocular traumatisms may result in loss of vision. Our previous work showed that therapeutic hypothermia prevents retinal damage caused by traumatic neuropathy. We also generated and characterized small molecules that elicit the beneficial effects of hypothermia at normal body temperature. Here we investigate whether one of these mimetic molecules, zr17-2, is able to preserve the function of eyes exposed to trauma.

**Methods:** Intraorbital optic nerve crush (IONC) or sham manipulation was applied to Sprague-Dawley rats. One hour after surgery, 5.0 µl of 330 nmol/L zr17-2 or PBS, as vehicle, were injected in the vitreum of treated animals. Electroretinograms were performed 21 days after surgery and a- and b-wave amplitude, as well as oscillatory potentials (OP), were calculated. Some animals were sacrificed 6 days after surgery for TUNEL analysis. All animal experiments were approved by the local ethics board.

**Results:** Our previous studies showed that zr17-2 does not cross the blood-ocular barrier, thus preventing systemic treatment. Here we show that intravitreal injection of zr17-2 results in a very significant prevention of retinal damage, providing preclinical support for its pharmacological use in ocular conditions. As previously reported, IONC resulted in a drastic reduction in the amplitude of the b-wave (*p* < 0.0001) and OPs (*p* < 0.05), a large decrease in the number of RGCs (*p* < 0.0001), and a large increase in the number of apoptotic cells in the GCL and the INL (*p* < 0.0001). Interestingly, injection of zr17-2 largely prevented all these parameters, in a very similar pattern to that elicited by therapeutic hypothermia. The small molecule was also able to reduce oxidative stress-induced retinal cell death *in vitro*.

**Discussion:** In summary, we have shown that intravitreal injection of the hypothermia mimetic, zr17-2, significantly reduces the morphological and electrophysiological consequences of ocular traumatism and may represent a new treatment option for this cause of visual loss.

## 1 Introduction

Trauma to the ocular globe and the optic nerve is a relatively common finding in daily scenarios that may include motor vehicle accidents, house falls, work- and sport-related injuries, or some cases of child abuse (Singman et al., 2016). In addition, it is a very common injury in combat situations as a result of explosive blasts (Flanagan et al., 2020). Traumatic optic neuropathy may result in axon degeneration and death of the retinal ganglion cells (RGC) through anterograde (You et al., 2012) or retrograde (Hendrickson et al., 2015; Dinkin 2017) mechanisms, resulting in severe loss of vision. Although several experimental protocols are being tested (Morgan-Warren et al., 2013; Mysona et al., 2017; Mesentier-Louro et al., 2019; Nascimento-Dos-Santos et al., 2020), no effective treatment is currently available for these patients.

Therapeutic hypothermia consists in reducing the body (or a particular organ´s) temperature to obtain medical benefits. Its usefulness has been proven in managing several pathologies such as neonatal asphyxia (Cornette 2012; Rey-Funes et al., 2021), coronary artery bypass surgery (Sirvinskas et al., 2014), neurodegeneration after cardiopulmonary resuscitation (Arrich et al., 2012), stroke (Ikeda et al., 2012; Minnerup et al., 2012; Lyden et al., 2014; Rogers et al., 2014), or ocular traumatisms (Rey-Funes et al., 2017), among others. However, despite these accomplishments, the clinical implications of therapeutic hypothermia in central nervous system (CNS) injury remain controversial. For instance, randomized controlled trials for traumatic brain injury (Clifton et al., 2001; Clifton et al., 2011; Kramer et al., 2012) and spinal cord injury (Martirosyan et al., 2017) failed to show beneficial effects. This could probably follow from the difficulties of cooling inner organs, and specifically the brain and spinal cord, which may require extracorporeal blood refrigeration (Lyden et al., 2014; Sirvinskas et al., 2014). In addition, exposure to cold temperatures may have a negative impact on the patients. Reported adverse effects of therapeutic hypothermia include extreme hypothermia, bradycardia, hypoglycemia, sepsis, skin necrosis, pulmonary hypertension, and systemic hypotension, among others (Saw et al., 2019).

The mechanism by which therapeutic hypothermia prevents cellular damage has been classically ascribed to a reduction of neuroinflammation and alleviation of metabolic demands (Zeevalk and Nicklas 1993; Martirosyan et al., 2017). However, in the last few years, special attention is being dedicated to the so-termed cold-shock proteins (CSP). These are a group of proteins that instead of reducing their expression when cells are subjected to hypothermia, as is the common behavior for most proteins, they increase their relative presence. Two main CSPs have been described in mammals: RNA-binding motif protein 3 (RBM3) and cold inducible RNA-binding protein (CIRBP). These proteins bind to cellular mRNAs and regulate their half-life, increasing the expression of their target proteins, which usually are involved in survival functions within the cell (Wellmann et al., 2004; Lleonart 2010; Wellmann et al., 2010; Liu et al., 2013). The neuroprotective effects of CSPs have been clearly demonstrated (Chip et al., 2011; Avila-Gomez et al., 2020) and they are expressed throughout the CNS (Liu et al., 2010), including the retina (Larrayoz et al., 2016).

In order to solve some of the problems associated to therapeutic hypothermia, namely the difficulty to reach inner organs and the potential adverse effects of hypothermia, we have recently identified small molecules that are able to induce some of the consequences of hypothermia, such as increasing CIRBP expression, but without reducing body temperature (Coderch et al., 2017). This was done through a combination of *in silico* virtual screening of small molecules against the 3D structure of CIRBP, *in vitro* testing on retinal cell line R28, and *in vivo* analysis on rat tissues. From the several small molecules able to induce CIRBP expression, zr17-2 was chosen as the most promising since it was the most efficient in promoting CIRBP expression and was water-soluble. Interestingly, zr17-2 was shown to be unable to cross the blood-brain and the blood-eye barriers (Coderch et al., 2017).

Therefore, the aim of the present study was to check whether the intravitreal injection of zr17-2 is able to prevent retinal damage caused by optic nerve trauma, in a similar manner as therapeutic hypothermia does (Rey-Funes et al., 2017).

## 2 Materials and methods

### 2.1 Traumatic injury model and zr17-2 intravitreal injection

Male young (8 week old) Sprague-Dawley albino rats (*n* = 24) with genetic quality and sanitary certification from the animal facility of our Institution were cared for in accordance with the guidelines published in the ARVO Statement for the Use of Animals in Ophthalmic and Vision Research. The treatments described below were approved by the Ethical Committee of CICUAL: “Comité Institucional para el Uso y Cuidado de Animales de Laboratorio” (Resolution number: RESCD-2022–1977-E-UBA-DCT#FMED), Facultad de Medicina, Universidad de Buenos Aires, *Argentina*. Animals were kept under standard laboratory conditions, with light/dark cycles of 12/12 h, and food and water were given *ad libitum*.

**FIGURE 1:**
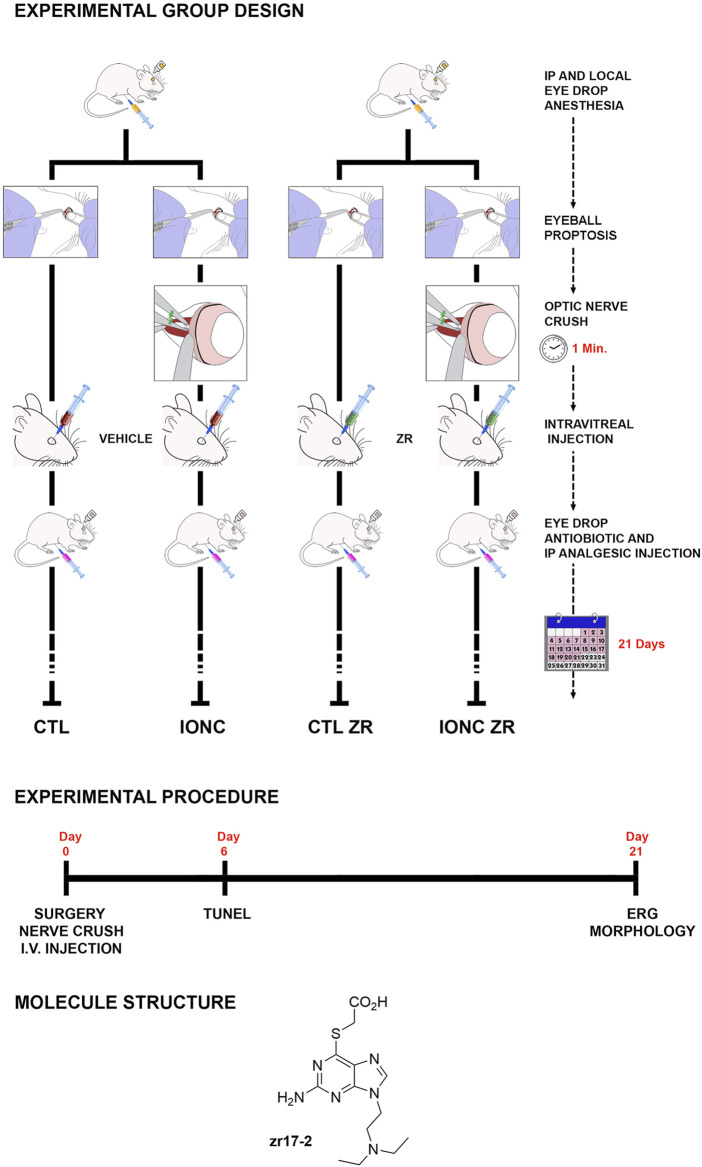
Schematic drawing of the different treatments generating the four experimental groups of eyes used in this study: i) Control eyes (CTL) sham operated and injected with PBS, ii) Traumatism control (intraorbital optic nerve crush, IONC) and injected with PBS, iii) Control ZR (CTL ZR) sham operated and injected with zr17-2, iv) Treated traumatism IONC operated and injected with zr17-2 (IONC ZR). The experimental procedure timeline is indicated, as well as the small molecule structure.

The small molecule zr17-2 was initially synthesized in house in the search of new CK2 inhibitors. This compound is a purine derivative (Figure 1) which mimics the adenine present in ATP, and therefore may act as an ATP competitive kinase inhibitor. Although docking studies predicted high affinity for CK2, a radiometric enzymatic assay revealed that the compound was inactive against this enzyme (Maslyk et al., 2010). Some years later, compound zr17-2 was identified as a hypothermia mimetic using a High Throughput Virtual Screening (HTVS) on diversity set IV of the NCI and fifteen molecules of our database (Coderch et al., 2017).

The intraorbital optic nerve crush (IONC) protocol was performed as described (Rey-Funes et al., 2017), with slight modifications. Briefly, rats were anesthetized with 2% Isofluorane (Baxter, Deerfield, IL) using an E-Z anesthesia vaporizer system (Euthanex, Corp, Palmer, PA). The left eye was subjected to IONC whereas the right eye was sham operated, as follows: A small incision was performed in the superior temporal quadrant of the left eye and eyeball proptosis was performed to provide access to the optic nerve. The optic nerve was crushed with forceps for 60 s at 1.5 mm from the ocular globe, and the incision was closed with suture. The right eye was sham-operated: the optic nerve was exposed but not crushed. One hour after surgery, animals were randomly divided in two groups, and received a 5.0 µl intravitreal injection of either 330 nmol/L zr17-2 or phosphate buffered saline (PBS), as vehicle (Figure 1). For the intravitreal injection, rats were anesthetized again and a local anesthetic (0.5% proparacaine) and a mydriatic solution (5% phenylephrine, 0.5% tropicamide) were applied to each eye. A 21G needle was inserted through the pars plana and the needle of a Hamilton syringe was introduced through the larger needle. When the blunt end of the Hamilton needle was seen protruding into the vitreous chamber, a 5.0 µl of the intended solution was slowly injected. The Hamilton and the 21G needles were simultaneously removed. To prevent any potential infection or pain resulting from the procedure, rats were treated with antibiotics and analgesics (5 mg/kg Tramadol, i. p., every 3 days, plus topical application of ciprofloxacin 0.3 g and dexamethasone 0.1g) during the week following surgery.

Therefore, this procedure generated four experimental eye groups: i) Control eyes (CTL, *n* = 9) sham operated and injected with PBS; ii) Traumatism control (IONC, *n* = 9) IONC operated and injected with PBS; iii) Control ZR (CTL ZR, *n* = 9) sham operated and injected with zr17-2; and iv) Treated traumatism (IONC ZR, *n* = 9) IONC operated and injected with zr17-2 (Figure 1). Twenty-one days after surgery, these animals were subjected to electroretinography (see below) and sacrificed by an anesthetic overdose. Then, the eyes were used for histological analyses (see below).

For TUNEL analysis, some additional animals of the four experimental groups (*n* = 3 eyes per group) were sacrificed 6 days after surgery.

### 2.2 Electroretinograms

Twenty-one days after surgery, rats (*n* = 9 per experimental group) were subjected to electroretinography, as described (Roth et al., 2016). Briefly, after overnight adaptation in the dark, rats were anesthetized under dim red illumination. An ophthalmic solution of 5% phenylephrine hydrochloride and 0.5% tropicamide (Fotorretin, Poen, Buenos Aires, *Argentina*) was used to dilate the pupils. Rats were placed facing the stimulus at a distance of 25 cm in a highly reflective environment. Scotopic electroretinograms (ERG) were recorded from both eyes simultaneously and 20 responses were collected to flashes of unattenuated white light (1 m, 1 Hz) from a photic stimulator set at maximum brightness. The registered response was amplified (9 cd s/m^2^ without filter), filtered (1.5-Hz low-pass filter, 500Hz high-pass filter, notch activated), and averaged (Akonic BIO-PC, Buenos Aires, *Argentina*). The a-wave was measured as the difference in amplitude between the recording at onset and the trough of the negative deflection and the b-wave amplitude was measured from the trough of the a-wave to the peak of the b-wave. To calculate oscillatory potentials (OP), the same photic stimulator was used with filters of high (300 Hz) and low (100 Hz) frequency. The amplitudes of the OPs were estimated by using the peak-to-trough method.

### 2.3 Histology and TUNEL assay

At the end of the electroretinography, rats were deeply anaesthetized (300 mg/kg ketamine, Imalgene, Merial Laboratorios, Barcelona, Spain, +30 mg/kg xylazine, Xilagesic, Proyma Ganadera, Ciudad Real, Spain), and intracardially perfused with 4% paraformaldehyde in PBS. The eyes were postfixed in the same fixative for 24 h at 4°C, paraffin embedded, and sectioned (3 µm thick). Sections were stained with hematoxylin and eosine. Additional retinas, taken from animals sacrificed 6 days after surgery (n = 3 animals per experimental group), were stained for terminal deoxynucleotidyl transferase dUTP nick end labeling (TUNEL) with the *In Situ* Cell Death Detection Kit, POD (Roche, Basel, Switzerland), following manufacturer´s instructions. Images were captured using an optic microscope (BX40, Olympus Optical Corporation, Tokyo, Japan), fitted with a digital camera (390CU 3.2 Megapixel CCD Camera, Micrometrics, Spain), and the image software Micrometrics SE P4 (Standard Edition Premium 4, Micrometrics, Spain). Before assays, care was taken in selecting anatomically matching areas among animals for an accurate analysis. To avoid variations in the quantification process, all the images for the same technique were obtained the same day and under the same light and contrast conditions. The number of RGCs per 1,000 µm of retinal length (3 eyes, seven fields per eye, for a total of 21 fields per group) or TUNEL-positive cells per ×25 objective microscopic field (3 eyes, six fields per eye, for a total of 18 fields per group), was estimated in each experimental group, as previously reported (Rey-Funes et al., 2017).

### 2.4 Cell toxicity assay

Immortalized rat retinal precursor cell line R28 was a gift from Dr. Patricia Becerra (National Eye Institute, NIH, Bethesda, MD, United States). R28 cells were cultured in DMEM/F12 medium supplemented with 5% fetal bovine serum (Invitrogen, Alcobendas, Spain) at 37°C in an atmosphere containing 5% CO_2_. Cells were seeded in 96-well plates at a density of 5 × 10^3^ cells per well, allowed to attach for 24 h, and exposed to different concentrations of zr17-2 and aluminum maltolate [Al (mal)_3_], as reported (Bobadilla et al., 2021). Cell number was estimated using the Cell Titer 96 Aqueous One Solution Cell Proliferation Assay (Promega, Madison, WI, United States), following manufacturer’s instructions. Twenty µl of MTS reagent (3-(4,5-dimethylthiazol-2-yl)-5-(3-carboxymethoxyphenyl)-2-(4-sulfophenyl)-2H-tetrazolium) were added to each well and incubated for 4 h. Absorbance was examined at 490 nm using a microplate reader (POLARstar Omega, BMG Labtech, Ortenberg, Germany).

### 2.5 Statistical analysis

All data were analyzed with GraphPad Prism five software and were considered statistically significant when *p* < 0.05. Values are expressed as means ± SD. All data sets were evaluated for normality (Shapiro-Wilk) and homoscedasticity (Levene). Normally distributed data were evaluated by ANOVA followed by the Dunnet’s *post hoc* test while data not following a normal distribution were analyzed with the Kruskal–Wallis test followed by the Mann-Whitney *U* test.

## 3 Results

### 3.1 Small molecule zr17-2 restores electroretinogram patterns

Experimental animals were randomly divided into two groups (vehicle vs. treatment with zr17-2) and their left eye was subjected to IONC whereas their right eye was sham operated. This procedure generated four groups of eyes. All animals were examined daily after surgery and none of them experienced any adverse reaction to either the small molecule or the surgical procedure.

**FIGURE 2:**
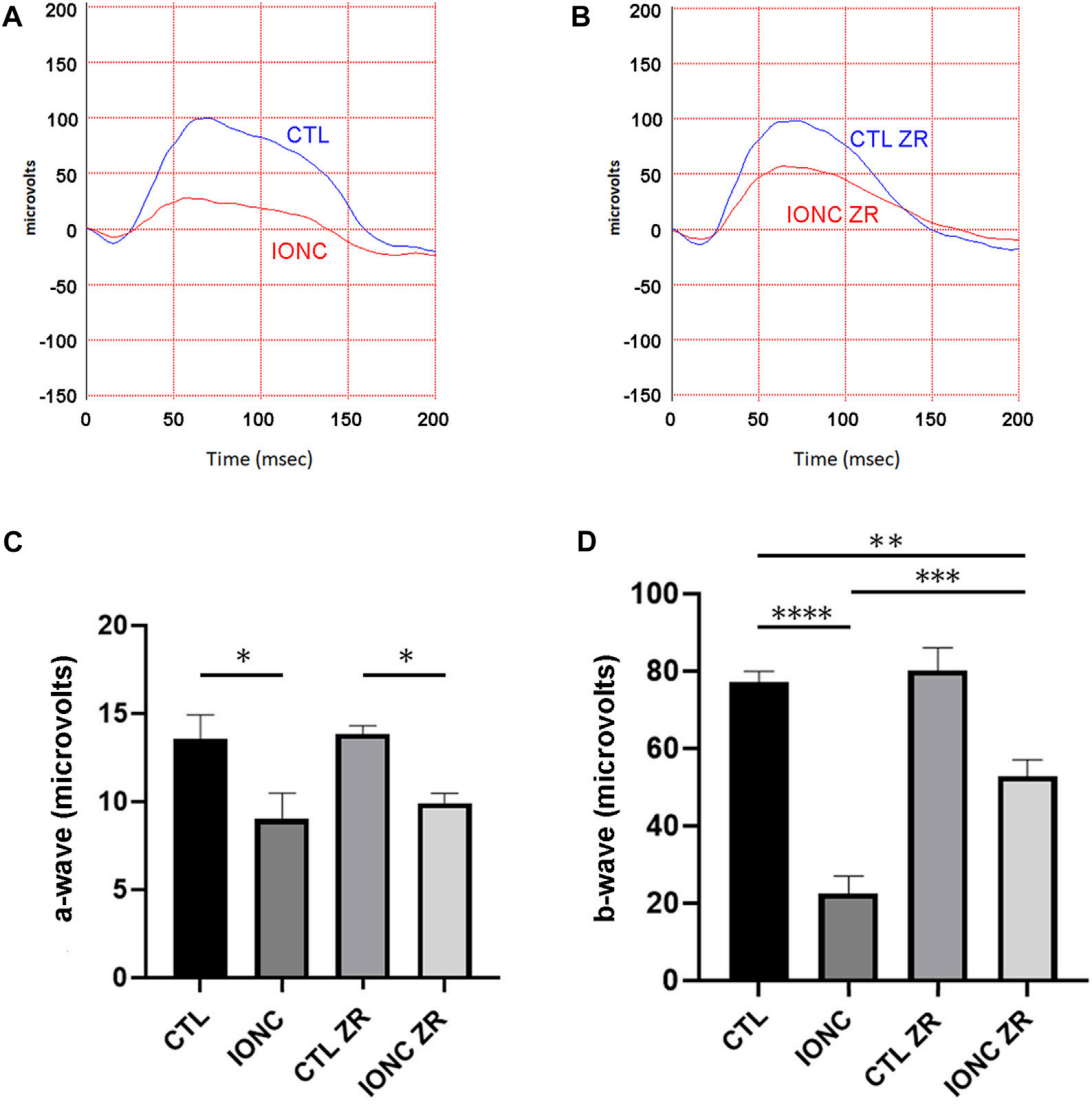
Electroretinograms and quantification of the a- and b-waves on the four experimental groups. Representative electroretinograms of vehicle- **(A)** and zr17-2-treated **(B)** animals subjected to IONC (red lines) or sham-operated (blue lines). The a- **(C)** and b-waves **(D)** were quantified. Bars represent the mean ± SD of all animals (*n* = 9 per experimental group). Asterisks represent statistically significant differences (ANOVA). *: *p* < 0.05; **: *p* < 0.01; ***: *p* < 0.001; ****: *p* < 0.0001.

**FIGURE 3:**
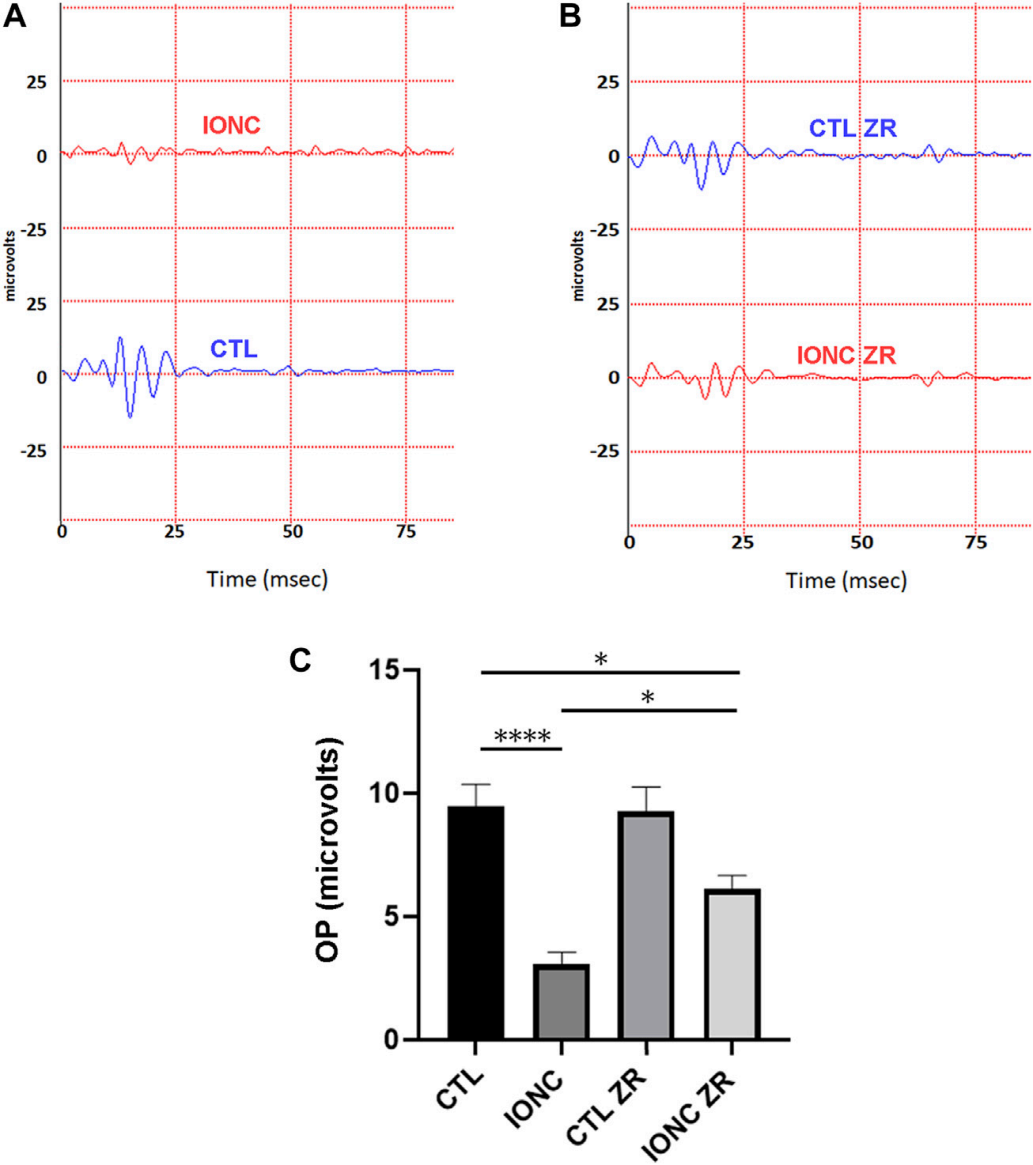
Electroretinograms and quantification of oscillatory potentials on the four experimental groups. Representative electroretinograms of vehicle- **(A)** and zr17-2-treated **(B)** animals subjected to IONC (red lines) or sham-operated (blue lines). Oscillatory potentials of the four groups were quantified **(C)**. Bars represent the mean ± SD of all animals (*n* = 9 per experimental group). Asterisks represent statistically significant differences (ANOVA). *: *p* < 0.05; ****: *p* < 0.0001.

Twenty-one days after surgery, all animals were subjected to scotopic ERG. In agreement with previous reports (Rey-Funes et al., 2017), the IONC procedure resulted in a significant reduction of the a-wave (Figures 2A–C), the b-wave (Figures 2A,B,D), and the OPs (Figure 3). Injection of zr17-2 in sham-operated animals did not modify retinal electrophysiology (Figures 2C,D; Figure 3C). On the other hand, injection of zr17-2 in IONC-damaged eyes resulted in a significant recovery (*p* < 0.001 and *p* < 0.05, respectively) of the b-wave (Figure 2D) and oscillatory potentials (Figure 3C).

### 3.2 Small molecule zr17-2 prevents loss of retinal ganglion cells

**FIGURE 4:**
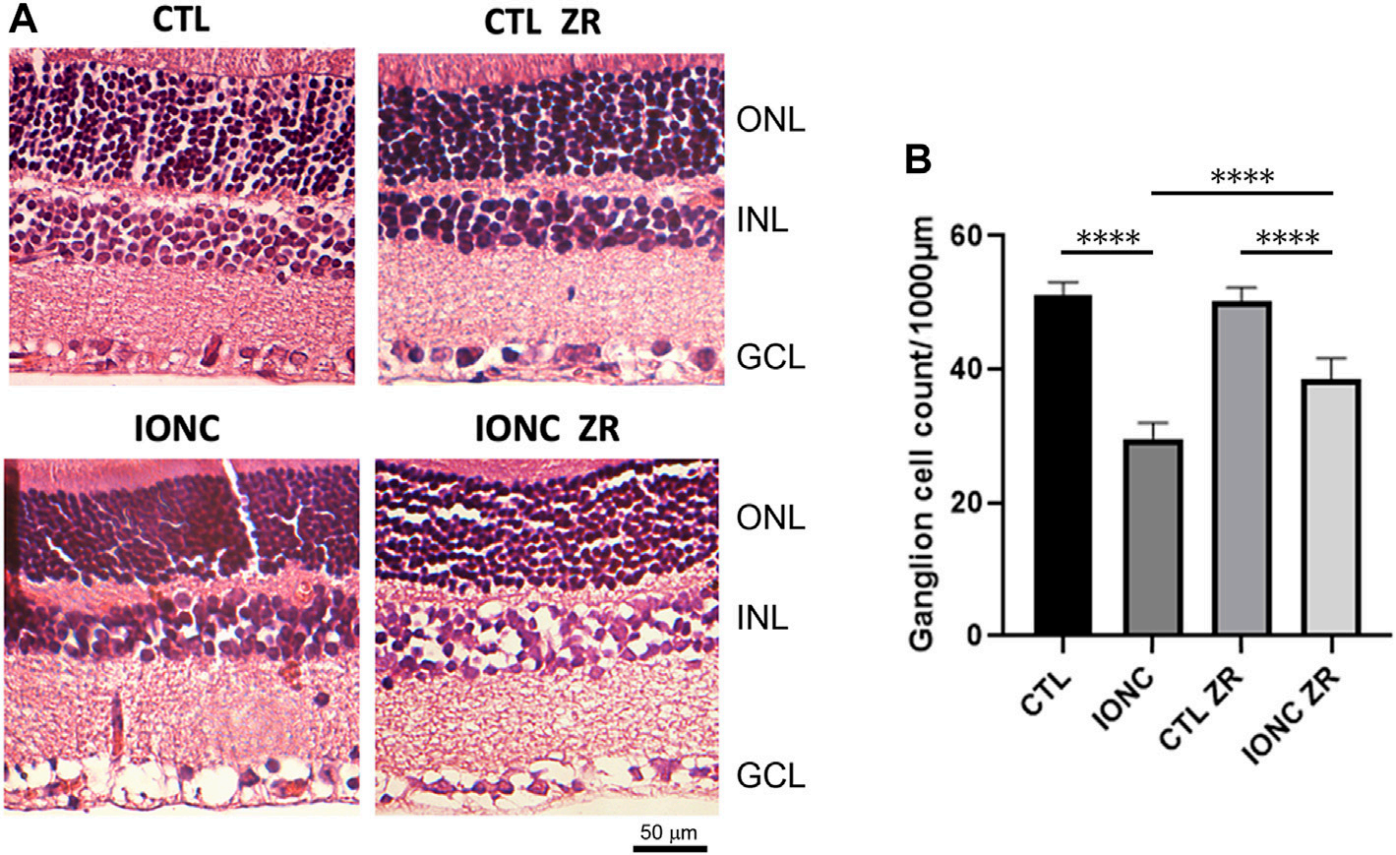
Anatomical structure of the retinas on the four experimental groups. Representative sections of the retina of treated animals stained with hematoxylin and eosin **(A)**. The nuclear layers of the retina are indicated: outer nuclear layer (ONL), inner nuclear layer (INL), and ganglion cell layer (GCL). Size bar = 50 µm. Quantification of the number of ganglion cells per 1,000 µm **(B)**. Bars represent the mean ± SD of all animals (*n* = 21 fields per experimental group). Asterisks represent statistically significant differences (ANOVA). ****: *p* < 0.0001.

Our morphological data show that optic nerve traumatism (IONC) translates into a major loss (*p* < 0.0001) of RGCs in the GCL (Figure 4). Injection of zr17-2 resulted in a significant (*p* < 0.0001) prevention of such a loss, although these values were still lower than the controls (Figure 4). As in the electroretinograms, application of zr17-2 in sham-operated eyes did not modify the number of RGCs (Figure 4).

### 3.3 Small molecule zr17-2 reduces IONC-mediated apoptosis in the retina

**FIGURE 5:**
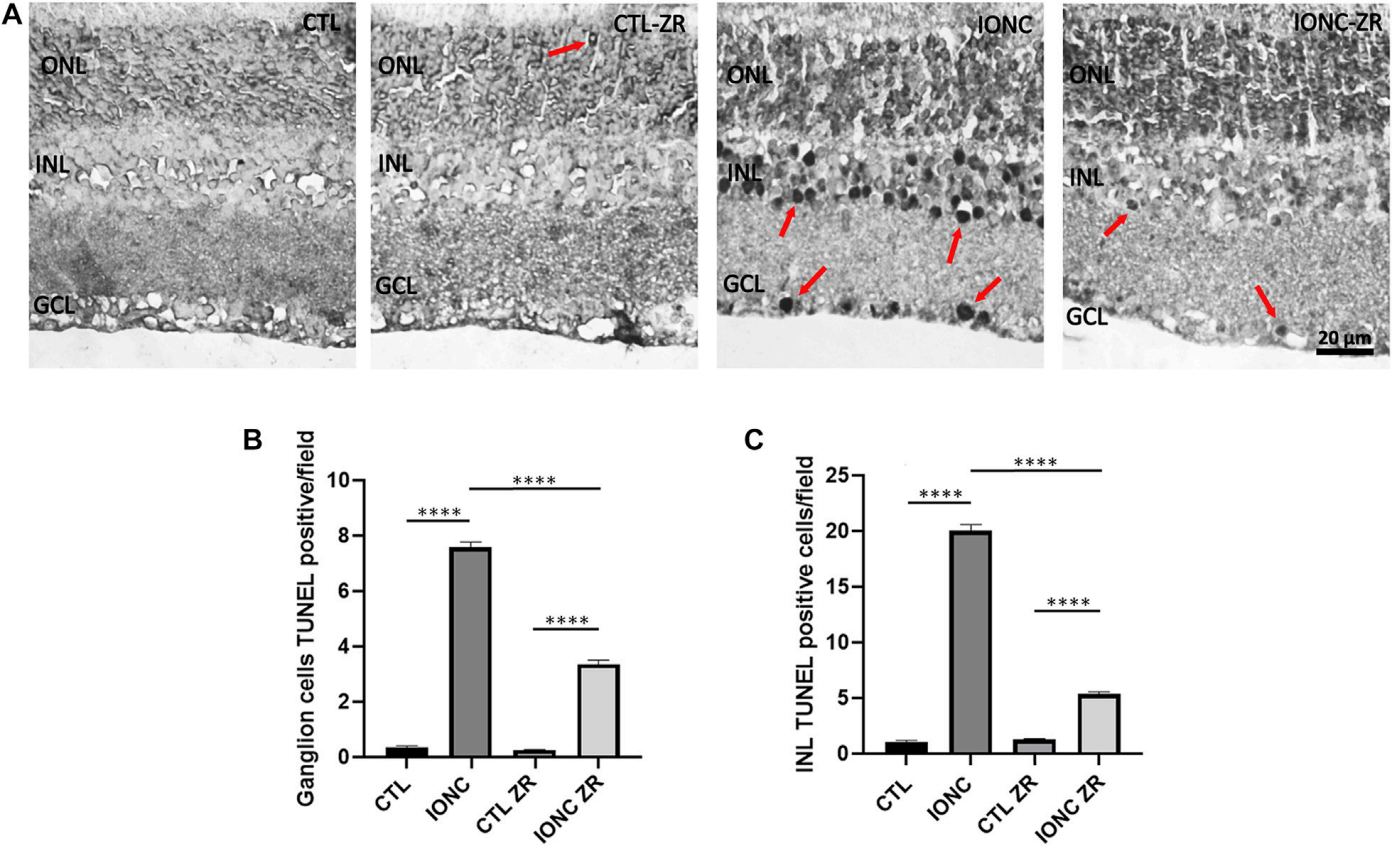
TUNEL positive cells on the four experimental groups. Representative images of retinas from animals in the four experimental groups taken 6 days after surgery **(A)**. The nuclear layers of the retina are indicated: outer nuclear layer (ONL), inner nuclear layer (INL), and ganglion cell layer (GCL). TUNEL positive cells (arrows) were found mainly in the GCL and the INL. Size bar = 20 μm. Quantification of the results for the GCL **(B)** and the INL **(C)** is shown as histograms. Bars represent the mean ± SD of all animals (*n* = 18 fields per experimental group). Asterisks represent statistically significant differences (ANOVA). ****: *p* < 0.0001.

As expected, rats that were subjected to the IONC treatment presented a very high number of TUNEL-positive cells in both the GCL and the INL when compared with that of the control animals (*p* < 0.0001) (Figure 5). Treatment with zr17-2 greatly reduced the number of apoptotic cells (*p* < 0.0001), although they did not reach the basal number (*p* < 0.0001). Interestingly, zr17-2 did not increase the number of TUNEL-positive cells in the eyes that were not subjected to IONC, indicating that this small molecule is not toxic (Figure 5). Sporadically, some TUNEL-positive cells could be seen in the ONL (Figure 5A).

### 3.4 Small molecule zr17-2 reduces oxidative stress-mediated retinal cell death *in vitro*

**FIGURE 6:**
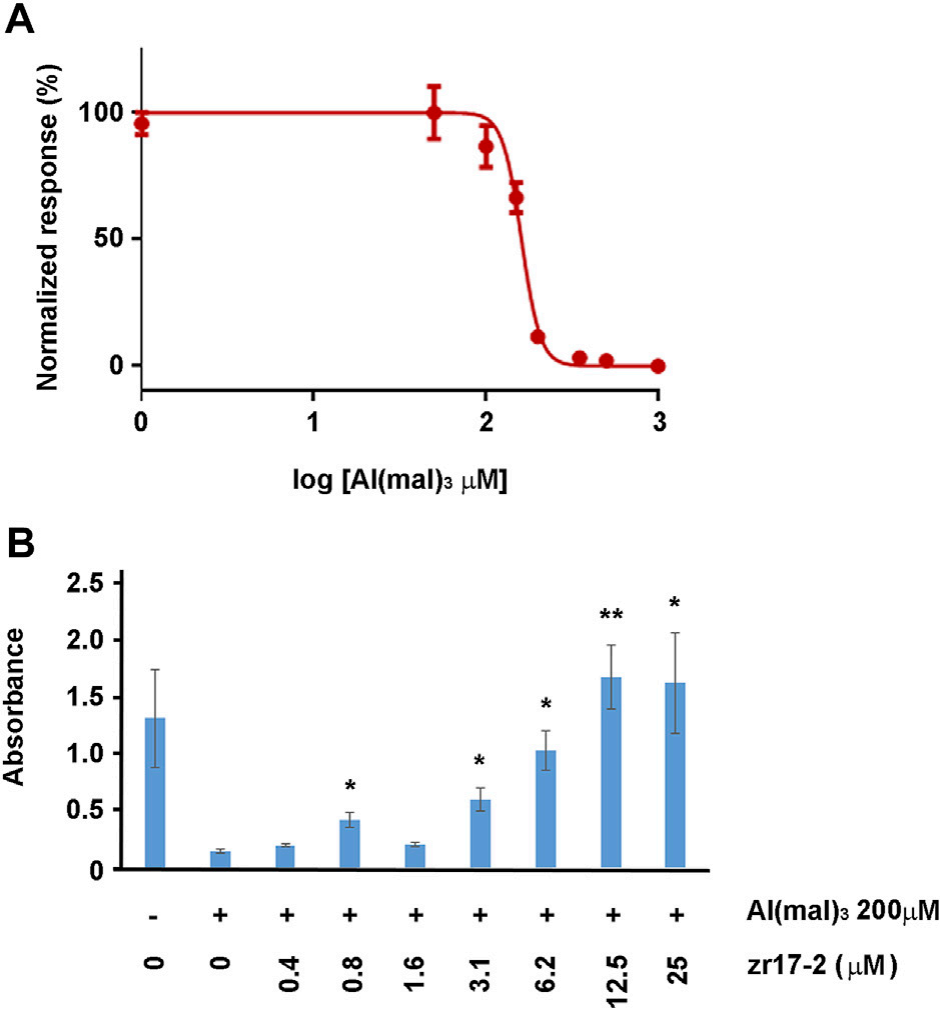
R28 cells responses to Al (mal)_3_ and zr17-2. First, a toxicity assay was performed to determine the IC_50_ of Al (mal)_3_ on R28 **(A)**. Then, cells were exposed to 200 µM Al (mal)_3_ and increasing concentrations of zr17-2. Aluminum maltolate drastically reduced cell number whereas zr17-2 significantly prevented cell death in a dose-dependent fashion **(B)**. Bars represent the mean ± SD of all wells (n = 8 per experimental condition). Asterisks represent statistically significant differences with the Al (mal)_3_ treatment (second bar) (ANOVA). *: *p* < 0.05; **: *p* < 0.01.

To confirm whether zr17-2 was able to work *in vitro*, immortalized rat retinal precursor cell line R28 was treated with Al (mal)_3_, an oxidative stress inducer. Apparently, this is the first time that R28 cells are exposed to this substance (a Pubmed search came up empty), so we performed a concentration-dependent toxicity assay to determine the IC_50_ of Al (mal)_3_ in this cell line. After 3 days in culture, the IC_50_ was calculated as 160.7 µM (Figure 6A). A concentration of Al (mal)_3_ of 200 µM, a little higher than the IC_50_, was chosen for further experiments. When a combination of Al (mal)_3_ and zr17-2 was applied to the cells, a significant dose-dependent prevention of cell death was observed (Figure 6B).

## 4 Discussion

In this study we have shown that the hypothermia mimetic small molecule, zr17-2, was able to significantly reduce several morphological and physiological markers of vision loss caused by traumatism to the optic nerve. This is the first time that a hypothermia mimetic is shown to recapitulate the beneficial effects of hypothermia in an *in vivo* model.

Although the initial manuscript describing zr17-2 showed that systemic injection of this molecule does not increase CIRBP expression in the brain or the eye (Coderch et al., 2017), we have shown here that intravitreal injection results in beneficial effects for the retina. Therefore, if we want to use the protective properties of zr17-2 in the eye or the CNS we will need to choose carefully the route of administration.

All animals were checked daily and no adverse effects due to either the molecule or the surgical procedure were ever observed. In addition, TUNEL analysis showed no increase on the number of apoptotic cells in the sham-operated retinas treated with zr17-2. This indicates that the small molecule is not toxic, at least at the concentration used, and is safe to be employed *in vivo*. Nevertheless, before these results could be translated into humans, full preclinical and clinical studies must be conducted.

Previous studies had shown that IONC results in a reduction of amplitude for the b-wave and the OPs of the ERG, an important diminution on the number of RGC in the GC layer, and a large increase on apoptotic cells in the same location. These morphological and physiological hallmarks coincided with a serious loss of vision as measured with a behavioral test (Rey-Funes et al., 2017). Our current data confirm these previous results. Furthermore, the presence of a large number of apoptotic cells in the GCL and INL parallels the modification of the b-wave (which accounts for the action potential of the RGCs) and of the OPs (which are produced by the cells of the INL) (Jung et al., 2015). Nevertheless, the important message here is that zr17-2 was able to significantly reduce all these parameters (ERG amplitude, number of RGCs, number of apoptotic cells in the retina) in a manner that is comparable to the effects caused by regular hypothermia (Rey-Funes et al., 2017).

There was also a small reduction in the amplitude of the a-wave caused by IONC. This may be due to a retrograde influence through the retinal circuit. Not many apoptotic cells were observed among the photoreceptors in the ONL, which are responsible for the a-wave. In any case, the a-wave reduction was not prevented by injection with zr17-2.

The dose of zr17-2 that was injected into the eyes was calculated based on previous *in vivo* experiments with this molecule (Coderch et al., 2017) and factoring in the eye volume. This particular concentration was able to significantly reduce the morphological and physiological parameters of retinal degeneration, but was not able to bring them close to the control values. Future experiments would explore whether increasing the dose of zr17-2 translates into a stronger protection.

It has been suggested that the mechanism of action by which zr17-2 modulates CIRBP expression may be related with the blocking of a protease, which degrades CIRBP (Coderch et al., 2017). It has been shown that CIRBP binds to cellular mRNAs through a specific nucleotide pattern (Xia et al., 2012; Liu et al., 2013). In addition, upregulation of CSPs results in the elevation of antiapoptotic proteins such as BCL2 and a downregulation of proapoptotic proteins such as BAX, BAD, or BAK (Zhang et al., 2015; Wu et al., 2016). Thus, we can devise a scenario in which optic nerve trauma causes a retrograde signal to the RGCs encouraging them to initiate programmed cell death mechanisms, which would result in a massive apoptosis of RGCs a few days after injury, leading to important vision losses. If the eye receives an injection of zr17-2 within the proper time window (before the apoptosis mechanism reaches the point-of-no-return), the levels of CIRBP would rise, the mRNAs of genes involved in cell survival would increase their half-life, there would be more survival proteins in the cell, the apoptotic program would be stopped, and vision would be preserved. Nevertheless, we need to be cautious when interpreting these data. Although it has been demonstrated that zr17-2 induces an overexpression of CIRBP (Coderch et al., 2017), we cannot exclude at this time the possibility of off-target effects for this small molecule. Future full-genome transcriptomic studies will address this question.

This study shows that zr17-2 is very efficient in preventing ocular apoptosis in a context of optic nerve traumatism, but these results suggest that zr17-2 may be also useful in the treatment of CNS conditions where therapeutic hypothermia has been already proven efficacious. These include neonatal asphyxia (Cornette 2012), coronary artery bypass surgery (Sirvinskas et al., 2014), neurodegeneration after cardiopulmonary resuscitation (Arrich et al., 2012), or stroke (Ikeda et al., 2012; Minnerup et al., 2012; Lyden et al., 2014; Rogers et al., 2014). Additionally, considering that therapeutic hypothermia requires complex machinery that is not always available for the patients (Thomas et al., 2015), the really remarkable advantage of these promissory results is that the treatment with this small molecule would not require any expensive equipment.

We have also shown that zr17-2 can prevent oxidative stress-induced cell death *in vitro*. In addition to generating oxidative stress, Al (mal)_3_ exerts its toxicity through increasing ER stress (Rizvi et al., 2016) and neuronal ferroptosis (Zhu et al., 2022). Al (mal)_3_ is a potential environmental toxicant that may be involved in the generation of neurodegenerative diseases (Pan et al., 2020). Since oxidative and ER stress are at the basis of many eye- and brain-related pathologies (Khatri et al., 2018), this small molecule may find many additional applications in the field. In conclusion, zr17-2 works *in vivo* as an efficient hypothermia mimetic, reducing morphological and physiological hallmarks of retinal degeneration. Since zr17-2 works at regular body temperature and can reach even the deepest organs in the body, it may constitute a new and more effective way of applying the benefits of therapeutic hypothermia to a large number of human diseases and conditions.

## Data Availability

The raw data supporting the conclusion of this article will be made available by the authors, without undue reservation.
